# Probiotics in the Treatment of Radiotherapy-Induced Oral Mucositis: Systematic Review with Meta-Analysis

**DOI:** 10.3390/ph16050654

**Published:** 2023-04-27

**Authors:** Giuseppe Minervini, Rocco Franco, Maria Maddalena Marrapodi, Luca Fiorillo, Almir Badnjević, Gabriele Cervino, Marco Cicciù

**Affiliations:** 1 Multidisciplinary Department of Medical-Surgical and Odontostomatological Specialties, University of Campania “Luigi Vanvitelli”, 80121 Naples, Italy; 2 Department of Biomedicine and Prevention, University of Rome “Tor Vergata”, 00100 Rome, Italy; 3 Department of Woman, Child and General and Specialist Surgery, University of Campania “Luigi Vanvitelli”, 80121 Naples, Italy; 4 School of Dentistry, Department of Biomedical and Dental Sciences and Morphofunctional Imaging, University of Messina, via Consolare Valeria, 1, 98125 Messina, Italy; 5 Verlab Research Institute for Biomedical Engineering, Medical Devices and Artificial Intelligence, 71000 Sarajevo, Bosnia and Herzegovina; 6 Department of Biomedical and Surgical and Biomedical Sciences, Catania University, 95123 Catania, Italy

**Keywords:** microbiome, radiation-induced oral mucositis, probiotics

## Abstract

The inflammatory injury of the mucous membranes lining the digestive tract, from the mouth to the anus, is called mucositis. One of the intriguing and compelling new therapeutic modalities that has emerged in recent decades due to advances in our understanding of this condition’s pathophysiology is probiotics. The purpose of this meta-analysis is to evaluate the efficiency of probiotics in the treatment of chemotherapy-induced mucositis for head and neck malignancies; a literature search was performed on PubMed, Lilacs, and Web of Science, and articles published from 2000 to 31 January 2023 were considered, according to the keywords entered. The term “Probiotics” was combined with “oral mucositis” using the Boolean connector AND; at the end of the research, 189 studies were identified from the search on the three engines. Only three were used to draw up the present systematic study and metanalysis; this meta-analysis showed that the treatment of mucositis with probiotics is an effective method, and the analysis of the results of these studies showed that the use of probiotics promoted a decrease in the severity of mucositis symptoms.

## 1. Introduction

Mucositis is an inflammatory injury to the digestive tract’s mucous membranes, from the mouth to the anus. Chemotherapy and/or radiotherapy are the most frequent causes due to their detrimental impact on mucosal cells that divide quickly, which typically have a low survival rate and might not be replaced immediately. Still, it can also occur less frequently due to oral problems, autoimmune disorders, local infections, allergic reactions to food or drugs, reactions to local infections, or deficiencies. The terms stomatitis and oral mucositis may occasionally be used interchangeably [1,2,3,4]. However, mucositis indicates a distinct process and broadly describes oral tissue inflammation [5,6,7,8]. Mucositis can manifest clinically as severe redness and/or blisters at the mouth level, or as nausea, vomiting, and diarrhea that may or may not be accompanied by pain. Mucositis is a prevalent and harmful illness. Its prevalence during standard chemotherapy is between 20 and 40%, and it may reach 80% in individuals undergoing high-dose chemotherapy (such as a conditioning regimen for hematopoietic stem cell transplantation or HSCT) [9]. Approximately 60% of patients who experience it use mammalian targets of rapamycin inhibitors such as everolimus and temsirolimus. However, practically every patient with head and neck cancer undergoing radiotherapy develops mucositis to some extent [10,11,12]. Patients receiving implant therapy also have a significant incidence of it (range, 19–65%; mean, 45%) [13]. The condition’s presence and severity can affect a patient’s prognosis, as well as dietary intake and overall quality of life, because it might necessitate a brief interruption, dose decrease, or suspension of localized and/or system interactions, or it might result in early dental implant failure [14]. However, treating symptoms (difficulty swallowing, etc.), and their side effects (such as malnutrition, infections, or hospitalization) may have a significant financial cost [15,16,17]. The use of probiotics is one of the interesting and promising new treatment approaches that has been developed in recent decades because of the advancements in our understanding of the pathophysiology of this ailment. However, the strength of the data is still insufficient. Thus, no firm recommendations can be given. Villa and Sonis established a five-stage model about 15 years ago to describe the complex biochemical mechanisms behind the onset of mucositis. The damage and death of basal epithelial cells and the release of endogenous mediators (initiation phase) are caused by an initiating event, such as reactive oxygen species produced by chemotherapy and/or radiotherapy, a mechanical trauma, or an infection. This phase supports the second stage of the process. The damage and death of basal epithelial cells and the release of endogenous mediators (initiation phase) are caused by an initiating event, such as reactive oxygen species produced by chemotherapy and/or radiotherapy, a mechanical trauma, or an infection. This phase supports the second stage of the process. The most important and clinically relevant phase is ulceration, which is caused by progressive mucosal damage. Through the direct and indirect (e.g., endotoxins mediated) stimulation of invading macrophages, bacteria inhabit ulcers and further increase the local inflammatory environment, extending the mucosal damage. As a result, when evaluating the function of probiotics, the microbiota and its interactions with the mucosal layer play a significant role. However, when it comes to the connection between mucositis and microbiota, we might face “the chicken or the egg” problem [18,19]. Few reports involving patients taking anticancer treatments and healthy controls show differing mucosal colonization. Still, it is possible that immunological dysregulation caused by cancer can encourage the colonization and overgrowth of native pathobionts. Contrarily, studies show changes in microbial composition during chemotherapy and radiation therapy treatments. By altering both mucus secretion and the appearance of the mucus layer, oncologic treatments are to blame for the breakdown of the oral mucous membrane’s natural barrier [20]. Conventional radiotherapy frequently results in hyposalivation. However, intensity-modulated radiotherapy (IMRT) has been linked to greater salivary flow preservation, oral microbiota stability, and a decreased incidence of mucositis and xerostomia. Probiotics may have a good impact on the integrity of the mucosa in several ways, but largely by displacing the harmful bacteria. Improved symbiont/pathobiont balance may increase mucosal barrier permeability, activate cytoprotective pathways in epithelial cells, reduce local inflammation, and influence innate and adaptive immunity [21]. Even if the balance between the local microbiota and the host is complicated and uncertain, it is in this setting that the use of probiotics makes sense [22]. A total of 80% of patients receiving 5-fluorouracil have been found to have gastrointestinal mucositis (5-FU). The medication used and the plan followed affect how often diarrhea caused by chemotherapy occurs. The weekly regimen of irinotecan and 5-FU bolus has been found to cause the most diarrhea, with 10% of patients developing grade 3 to 4 mucositis. After taking irinotecan at higher doses for a week and at reduced doses once weekly for about two weeks, late-onset diarrhea is possible. The oral and gastrointestinal mucosa’s microbiota is significantly adversely affected by chemotherapy and radiation. Oral mucositis is closely associated with bacteremia and sepsis due to *Escherichia coli*, *Pseudomonas aeruginosa*, and *Candida albicans*. The discovery that lozenges containing *Lactobacillus brevis* produced anti-inflammatory metabolites led to the advancement in how probiotics were hypothesized in order to combat the side effects of chemotherapy and/or radiotherapy. The production of arginine deiminase and sphingomyelinase by *L brevis*, which hydrolyzes platelet-activating factors known to be connected to oral mucositis in radiation therapy, was described. The quantity of arginine that can be converted into nitric oxide, a key mediator of inflammation, is decreased by arginine deiminase’s conversion of arginine to ammonia and citrulline. Furthermore, their attractiveness increased when it was shown that probiotics administered for oral mucositis had no significant side effects. However, this local oral benefit did not reach the intestines, as 14 of 16 experienced diarrhea. Cancer treatment boosts Enterobacteriaceae and Bacteroides while decreasing commensals such as *Bifidobacteria*, *Clostridium cluster XIVa*, and *Faecalibacterium prausnitzii* in the intestines. These modifications cause gut dysbiosis and aid in the emergence of mucositis, especially in the form of diarrhea and bacteremia. The idea that probiotics can lessen the adverse effects of chemotherapy and radiotherapy stems from negative changes in the intestinal microbiota. Probiotics may have a good impact on the integrity of the mucosa in several ways, but largely by displacing the harmful bacteria. Improved symbiont/pathobiont balance may increase mucosal barrier permeability, activate cytoprotective pathways in epithelial cells, reduce local inflammation, and influence innate and adaptive immunity [23]. The purpose of this study, through a thorough literature review, was to carefully evaluate the possible beneficial effects of probiotics in treating oral mucositis induced by radiotherapy and/or chemotherapy.

## 2. Materials and Methods

### 2.1. Eligibility Criteria

All documents were assessed for eligibility based on the following Population (including animal species), Intervention, Comparison, and Outcomes (PICO):

(P) Participants were patients with head and neck cancer treated with chemotherapy.

(I) The exposure included patients with head and neck cancer and oral mucositis treated with probiotics.

(C) The comparison consisted of head and neck cancer patients treated with a placebo.

(O) The outcome was to evaluate patients treated with probiotics and assess probiotic effectiveness in decreasing the severity of mucositis symptoms. Studies have staged mucositis before and after probiotic therapy through the Common Terminology Criteria for Adverse Events (CTCAE). The secondary outcome is to evaluate the influence of probiotics not only in treating mucositis but also in treating gastrointestinal problems resulting from radio/chemotherapy. The secondary purpose of this meta-analysis was to evaluate the possible adverse effects of probiotics and the effects of probiotics on increased immunity.

Only articles that included data from the intervention’s conclusion were included. The exclusion criteria were (1) studies on patients with other possible causes of mucositis (smoking, diabetes); (2) cross-over study design; (3) studies written in a language other than English; (4) full-text unavailability (i.e., posters and conference abstracts); (5) studies involving animals; (6) review articles; (7) case reports; (8) patients treated with probiotics and other drugs.

### 2.2. Search Strategy

Scientific databases were used in the execution of the review (PUBMED, WEB of SCIENCE, LILACS). The electronic search was conducted between 3 January 2000 and 31 January 2023. “Probiotics” and “oral mucositis” were used with the Boolean operator AND.

MESH (Medical Subjects Headings) was used to help with the web search (Table 1).

The *Cochrane Handbook for Systematic Reviews of Interventions and Preferred Reporting Items for Systematic Reviews (PRISMA)* criteria were followed in conducting this systematic review (PRISMA checklist, Supplementary Materials). Through the prism diagram, articles that met the keywords were selected through a manual search and search engines. After special filters, non-English-language articles were excluded, and reviews were excluded in the first screening phase, again through filters; in the second screening phase, only RCTs were considered. In addition, a manual search of the leading registered clinical trials from EMBASE, CENTRAL, and Clinicaltrial.gov was performed. On 14 January 2023, the systematic review protocol was entered into the International Prospective Register of Systematic Reviews (PROSPERO) under the provisory reference number CRD42023392691.

### 2.3. Data Extraction

Two reviewers (R.F. and G.M.) separately extracted data from the included studies using individualized data extraction on a Microsoft Excel sheet. A third reviewer was used to obtain consensus in cases of disagreement. The following information was taken: (1) first author; (2) publication year; (3) type of therapy; (4) sample; (5) criteria; (6) results.

### 2.4. Quality Assessment

Using the Cochrane risk-of-bias tool for randomized trials, version 2 (RoB 2), two reviewers evaluated the publications’ bias risk. Any discrepancy was handled with a third reviewer until an agreement was obtained.

### 2.5. Statistical Analysis

The pooled analyses were performed using Review Manager version 5.2.8 (Cochrane Collaboration, Copenhagen, Denmark; 2014). The efficacy of probiotics in treating mucositis during head and neck radiotherapy was evaluated and compared with a control group. The risk ratio between the two groups was measured. Heterogeneity among studies was assessed using the Higgins Index (I2) and the chi-square test and classified as follows: low heterogeneity (<30%), medium heterogeneity (30–60%), and high heterogeneity (>60%). The *p*-value was set at 0.05.

## 3. Results

### 3.1. Study Characteristics

**Figure 1:**
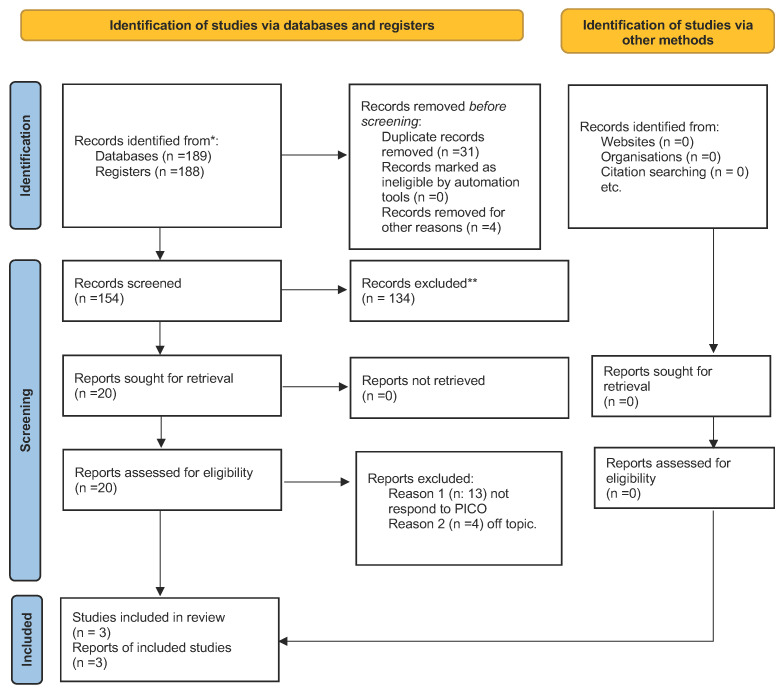
Prisma Flowchart. Prisma statement [24]. For more information, visit: http://www.prisma-statement.org/ accessed on 5 March 2023. * papers identified by search methods; ** papers removed because systematic reviews of the literature.

After searching the three search motors, 189 articles were selected. Using the exclusion criteria, articles not in English were automatically removed via the Boolean operator “NOT”. Specifically, one article from Web of Science and three from PubMed were deleted. Thirty-one articles were deleted because they were duplicates. During the first screening phase, 154 articles were considered, according to the inclusion criteria of clinical trials and randomized controlled trials, and so 134 articles were excluded. Therefore, 20 articles were published after this screening stage; the abstracts were read to assess for eligibility. According to the PRISMA 2020 flowchart in Figure 1, only four were chosen for this review. A total of 20 articles were excluded: 1 was a clinical study on rats, 13 were eliminated because they failed to meet the PICO criteria, and 3 articles were removed because they dealt with probiotics in the treatment of gastrointestinal diseases. According to the PICO model, three papers were chosen for title and abstract screening. From the Cochrane library, 125 clinical trials were selected with the exact search engine keywords. After manual selection with the reading of abstracts and titles, only one clinical trial was chosen for the meta-analysis. Therefore, three articles in total were selected for this meta-analysis. Of all articles on Embase and other search engines, the only eligible ones were equivalent. The studies ranged from 2018 to 2022, and were conducted in different parts of the world, including China, Italy, and India. Two hundred and thirteen patients were analyzed and divided, in a randomized, double-blind manner, into a study group in which a probiotic was administered, and a placebo group in which a similar substance was administered. Regarding the type of therapy, the three studies analyzed all considered patients who were to undergo radiotherapy and chemotherapy. All treatments and doses were the same in the analyzed studies. All mucositis patients were evaluated through the same universal rating scale, the National Cancer Institute’s Common Terminology Criteria for Adverse Events. The patient data were analyzed, and, for our meta-analysis, severe mucositis was defined as a grade above three and a lower grade was defined as not severe. Therefore, the patients were categorized, and the patients with a mucositis grade less than three were considered and were included as a study group in the table of the meta-analysis. Therefore, in De Sanctis’ study, 32 patients in the study group and 36 in the control were analyzed. In Jiang’s study, 64 patients in the study group and 35 in the control were analyzed. In Mirza’s study, 23 patients in the study group and 23 in the control were analyzed. The secondary outcome was assessed through the selection of the chosen articles, and two articles were taken up. The article by Mirza et al. evaluated the possible adverse effects of using probiotics, while the article by Jiang et al. evaluated the possible influence of probiotics on bone marrow for stimulating immunity.

### 3.2. Main Findings

**Figure 2:**
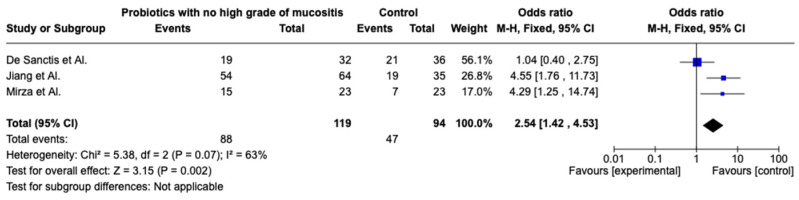
Forest plot of the meta-analysis [25,26,27].

This study’s objective was to test the impact of a probiotic combination on the severity of oral mucositis (OM), a common and preventable side effect of radiochemotherapy in patients with nasopharyngeal cancer who also receive concurrent radiochemotherapy. OM is a prevalent condition. The primary outcome was the occurrence of severe OM (grade 3 or above) in eligible patients (n = 99) with locally advanced nasopharyngeal cancer undergoing CCRT. Patients were randomly assigned (2:1) to receive a probiotic combination or placebo during radiochemotherapy. The concurrent radiochemotherapy (CCRT) group consisted of 35 patients, while the CCRT-P (CCRT + probiotic blend) group consisted of 58 patients. There was no discernible difference between the CCRT and the CCRT-P groups, and the treatment groups were evenly distributed. For the complete analysis set, the incidences of grade 0, 1, 2, and 3 OM were 0% and 10.94%, 0% and 51.56%, 54.29% and 21.88%, and 45.71% and 15.63%, respectively, in the placebo group and the probiotic combination group. For the per-protocol set, they were 0% and 12.07%, 0% and 55.17%, 54.29% and 17.24%, and 45.71% and 15.52%; this was consistent with the full-analysis set. Partial response rates were 4 of 35 (11.43%) and 7 of 58 (12.1%; *p* > 0.999); complete response rates were 31 of 35 (88.57%) and 51 of 58 (87.9%), thus being the opposite. The number of CD4+ T cells rose (76.59% vs. 52.85%), as did CD8+ T cells (62.94% vs. 29.76%) and CD3+ T cells (69.72% vs. 45.49%; *p* < 0.05; Figure 2). Additionally, the probiotic combination significantly increased hemoglobin (43.10% vs. 25.71%), lymphocyte ratio (8.62% vs. 2.94%), CD3+ T cells (18.92% vs. 0%), CD4+ T cells (8.33% vs. 0%), and CD8+ T cells (16.67% vs. 0%) in the CCRT-P group compared to the CCRT group [25]. Changing the microbiota in the oral cavity is a fascinating and challenging target. Radiation therapy with intensity modulation was used to treat all patients (IMRT). The macroscopic disease and low-risk regions received dose fractionation with specified total doses of 50–54 Gy and 68–70 Gy, respectively. Concomitant chemotherapy based on cisplatin was given by a weekly (40 mg/m^2^) or three-weekly (100 mg/m^2^) regimen. Before starting chemotherapy, enrolled patients were randomly assigned (1:1) to either the standard oral care regimen of sodium bicarbonate mouthwash (control arm) or LB *CD2* lozenges (intervention arm) using a computer-generated randomization list. The statistical analysis was conducted on a sample size of 68 patients (32 in the intervention arm and 36 in the control arm) as a result of 7 out of 75 patients being eliminated (4 were declared ineligible and were not randomly assigned, and 3 of the patients who were randomly assigned had too much missing data for the records that were available). Age, gender, body mass index, and clinical tumor stage were the same for the two population arms’ baseline patient characteristics. The incidence of severe OM was the same in the intervention and control groups during the RT course (40.6% and 41.6%, respectively, *p* = 0.974), according to the findings [26]. A parallel, randomized, double-blind, placebo-controlled trial was conducted on 46 patients who underwent radiation for head and neck cancers. *Bacillus clausii UBBC07* or regular treatment with placebo were randomly assigned to patients. *Bacillus clausii UBBC07* was administered orally twice daily for 30 days or until the entire radiation fraction had been administered. Using the CTCAE v.4.03 severity scale, the mucositis was graded. Fifty-two individuals were screened; two failed the screening, two declined to take part, and two stopped receiving therapy. The trial was completed by 46 individuals, of whom 43 were men and 3 were women. The median patient age was 55 (35–60). Compared to the placebo group, the test group’s median mucositis onset time increased significantly. The test group’s median time to mucositis onset was ten days compared to the control group’s eight days (*p* < 0.01). Compared to the placebo group, the median time for remission was considerably shorter in the test group (*p* < 0.05). The test group had a 12-day remission time, and the control group had a 14-day remission time. When comparing the test group to the placebo group, it was shown that there was a significantly lower incidence of higher-grade mucositis (grade III or above) (*p* < 0.05). In the test group, 8 out of 23 patients experienced greater grade mucositis, while 16 out of 23 patients in the placebo group experienced higher grade mucositis. In the test group, there were 15 respondents (65%) compared to 7 in the placebo group (30.4%). A total of 46 adverse events, including 20 in the test group and 26 in the placebo group, were identified in both research groups in addition to mucositis. The most frequent adverse events were xerostomia, dysgeusia, and dysphagia. Oral thrush, fever, and pneumonitis were the other incidents. There were four patients in the placebo group who experienced diarrhea, but none in the test group. Two participants in the placebo group needed standard therapy for their grade III ADRs. For feeding, a nasogastric tube was placed [27] (Table 2).

### 3.3. Metanalysis

The meta-analysis was conducted by random model effect because of the high heterogeneity (*I*^2^ = 63%) between the three included studies. The overall effect reported in the forest plot (Figure 2) shows that probiotics are an excellent aid for treating mucositis post-radio and chemotherapy for head and neck cancer (OR 2.54; 95% CI 1.42–4.53).

### 3.4. Quality Assessment and Risk of Bias

**Figure 3:**
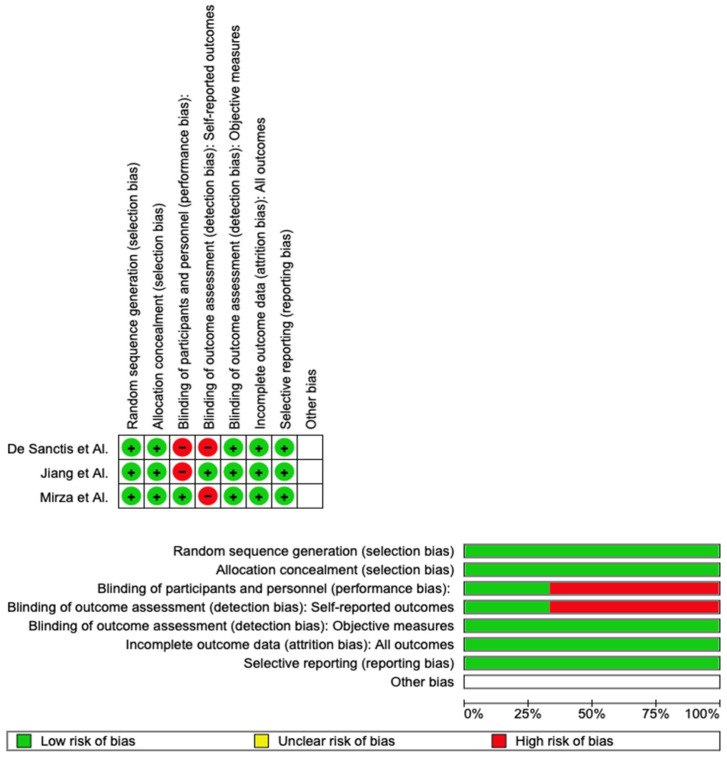
Risk of bias of the included studies [25,26,27].

Using RoB 2, the risk of bias was estimated and reported in Figure 3. Regarding the randomization process, 100% of the studies ensured a low risk of bias. However, 25% of the studies excluded a performance bias. Additionally, 100% of the included studies reported all outcome data, and 100% adequately excluded bias in the selection of reported outcomes, while 25% excluded bias in self-reported outcomes. Overall, all three were shown to have a low risk of experiencing bias.

## 4. Discussion

Jiang et al. showed that the probiotic combination dramatically decreased OM by raising the patients’ immunity. The findings revealed that only 15.52 per cent of patients in the CCRT-P group had grade 3 OM compared to 45.71 per cent of those in the CCRT group, supporting the probiotic combination’s preventive impact against OM. Because all recruited patients achieved comparable objective response rates, it should be highlighted that the probiotic blend did not affect the tumor response to CCRT. This outcome is equivalent to a study’s findings, which showed that one probiotic, *Lactobacillus brevis CD2*, had decreased the incidence of radio chemotherapy-induced OM (grades 3 and 4) in patients with head and neck squamous cell carcinoma who were receiving chemoradiation therapy [25,28]. The study of De Sanctis compares the incidence of OM between patients treated with sodium bicarbonate mouthwash and patients treated with LB *CD2* lozenges. The findings revealed that LB *CD2* did not affect the frequency of severe OM, taking into account the statistical restriction of the current trial (the targeted accrual was not met due to the absence of LB *CD2* supply) (intervention versus control arm: 40.6% versus 41.6%, respectively). Additionally, patients in the experimental group did not experience appreciable improvements in their quality of life or acute toxicities, including weight loss, discomfort, or dysphagia. Our study did not support the encouraging outcomes achieved by Sharma et al. According to Sharma et al., patients who received the LB *CD2* had a significantly reduced rate of grade 3 and 4 OM development than those who received the placebo (52 per cent against 77 per cent, *p* < 0.001) [26]. However, the idea that diseases such as OM might be linked to a change in the microbial makeup of the biofilm that colonizes the oral canal is garnering more and more interest. RCHT or the tumor itself may change the salivary bacterial flora, causing mucosal damage and modifying the levels of opportunistic bacteria that may become pathogenic in cancer patients who develop OM. Although few studies have focused on these conditions, there is also a lot of stress on the role of conditions at other oral sites, such as supra- and subgingival periodontitis.

Therefore, OM caused by RT is a complex process that needs to be approached from different therapeutic vantage points. Research into salivary microbiota is essential because it may offer an intriguing medicinal viewpoint.

Researchers could not corroborate the beneficial effects of *L. brevis CD2* in lowering the rate of grade 3–4 RT–induced OM in patients with HNC, even after considering that the early closure of patient accrual compromised the statistical power of the research. The study by De Sanctis et al. is the only one that does not corroborate the results of the others, probably because of either the premature closure of the study or the different types of probiotics. However, the most plausible hypothesis remains the early closure of the study.

In the Gupta study, oral mucositis affected every patient in both groups. Compared to the placebo group, we saw a substantial (*p* < 0.05) decline in high-grade mucositis in the test group. In the test group, there were eight patients with high-grade mucositis (34.8%), but in the placebo group, there were approximately sixteen patients with high-grade mucositis (69.6%). Compared to patients in the placebo arm, patients in the *LactoB. brevis CD2* lozenges arm also experienced a significantly decreased rate of high-grade oral mucositis (52 per cent vs. 77 per cent, respectively). In a different study by De Sanctis et al., it was discovered that the incidence of high-grade RIOM in the placebo and test groups was 40.6 per cent and 41.6 per cent, respectively. They concluded that *LactoB. brevis CD2* lozenges were ineffective in protecting head and neck cancer patients from radiation-induced oral mucositis. This meta-analysis showed that probiotics have protective effects on the oral mucosa. Although the heterogeneity of the meta-analysis is high, from the results, the improved development of probiotics in the treatment of mucositis post-radio and chemotherapy is evident [27].

Live microorganisms that help the general population and treat certain diseases by enhancing the microbial flora are called “probiotics”. The US Food and Drug Administration (FDA) has not yet approved any probiotic products for specific health conditions, despite the advantages demonstrated by clinical trials and animal models. FDA advice on INDs (Investigation New Drug) from 2013 states that probiotic products are treated as medications rather than as nutritional supplements if they are used to produce a physiological effect [29,30]. The National Institutes of Health and the FDA conducted research published in a report released by the Agency for Healthcare Research and Quality (AHRQ) in 2001. The report’s authors concluded that the current literature is not well equipped to confidently answer questions on the safety of probiotics in intervention studies [31]. Recurring infections, adverse metabolic processes, excessive immunological stimulation, gene transfer, obesity, skin issues, and gastrointestinal side effects are among the probiotics’ theoretical hazards. In patients, immunocompromised individuals, pregnant women, and persons with a probiotic propensity for translocation across the gut wall are among the populations that are at risk. Since all HSCT patients have immunosuppression and some HNC patients experience neutropenia because of anticancer therapy, extra vigilance and a thorough safety assessment are required before probiotics are administered. Sepsis is the side effect that has the worst effects on OM patients overall since it can interrupt therapy and potentially be fatal. However, there have been three separate reports of sepsis in HSCT patients brought on by Lactobacillus. Additionally, *lactobacillus rhamnosus*, isolated from patient blood, was found to be multi-antibiotic resistant, making management very difficult. Based on the available information, we are currently unable to determine whether it is safe or not for cancer patients to receive probiotics for OM because several variables are involved, including the probiotic strain, dosage, timing, and patient selection in the appropriate physical state. Probiotics’ method of protection is still unknown [32,33]. Despite the side effects, no side effects have been found regarding probiotics. According to earlier research, probiotics may regulate epithelial function, maintain the integrity of the mucosal barrier, boost host immunity, and prevent the colonization of harmful bacteria. According to animal tests, probiotics may shield epithelial cells by activating TLRs on macrophage surfaces and inducing a multicellular, adaptive immune signal cascade. In detail, peri cryptal macrophages’ TLR2 can bind to lipoteichoic acid (LTA), a probiotic from *Lactobacillus rhamnosus GG* (LGG), causing the chemokine CXCL12 to be produced. Then, mesenchymal stem cells (MSCs) that express COX-2 attach to CXCR4 on those cells, and this causes MSCs to become crypt epithelial stem cells in the lamina propria of the intestinal mucosa. Finally, PGE2 is released by MSCs to protect epithelial stem cells from radiation damage. The protective effects of LGG on epithelial cells were also eliminated in Myd88-/-, TLR2-/-, and COX2-/- animals, according to Ciorba’s findings [34,35]. We therefore hypothesize that the TLR-Myd88 pathway may be responsible for the protective action of probiotics. A study by Burdelya discovered that administering CBLB502, a polypeptide drug derived from Salmonella flagellin that binds to toll-like receptor 5 (TLR 5) and activates NF-B signaling, to mice before lethal total-body irradiation could protect them from developing gastrointestinal and hematopoietic acute radiation syndromes. Another experiment on animals showed that nonviable probiotics used the TLR9-Myd88 pathway to counteract dextran sodium sulfate-induced colitis, and their DNA was responsible for the protective benefits [36,37,38]. In conclusion, TLRs may be the essential site for probiotics’ success, and instead of using live probiotics, potent probiotic components such as LTA or DNA may be isolated and used. This can increase therapeutic efficacy while lowering any potential side effects, such as the sepsis discussed earlier. It was investigated further to see if oral probiotics could lessen the frequency of serious diarrhea (grade 2 or higher). Compared to the control group, oral probiotics could substantially reduce the incidence of oral mucositis and diarrhea in cancer patients receiving chemotherapy. Oral probiotic therapy was not associated with any deaths or negative outcomes. More specifically, seven studies found that patients could consume probiotics without risk, and two studies showed that patients had a high tolerance for them. This suggests that most probiotics, which are used to control gut microbiology, may be able to lessen the side effects of cancer treatment. Notably, Asian groups were the only ones to experience reduced chemotherapy-related side effects linked to probiotic use; neither European nor American populations did so. This may be related to the ethnic difference in that people in Western nations are accustomed to the daily consumption of probiotics, found in various fermented foods, leading to an immunologic tolerance to the probiotics. Extra clinical studies should be carried out to fully assess the inhibitory effects of oral probiotic use on the adverse effects caused by chemotherapy [39].

### Limitation of the Study

The limitation of this meta-analysis is due to the small number of patients and the different formulation of the probiotic mixture; in fact, as can be seen, this meta-analysis has high heterogeneity. Moreover, in addition to the small sample, other limitations of the study are the radiation dose used and the type of radio-chemotherapy the groups underwent. These factors may have affected the results. Another limitation lies in the duration of intake of the probiotics in addition to the recommended dose. However, despite these limitations, we can affirm the importance of taking probiotics as an adjuvant to the treatment of OM.

## 5. Conclusions

Although limited to the few studies in the literature, this meta-analysis showed the beneficial effects of probiotics on the oral mucosa and in enhancing the quality of life for patients with head and neck cancer. In addition to helping to resolve the symptomatic picture of oral mucositis, all studies have shown improvement in gastrointestinal symptoms due to radiation and chemotherapy with probiotics. In addition, all studies have shown a reduction in gastrointestinal symptoms. Therefore, probiotics may be helpful for the dual treatment of oral mucositis symptoms and gastrointestinal symptomatology due to radiation and chemotherapy. Apart from the study by De Sanctis et al., all studies have evaluated the beneficial effects of probiotics on the treatment and prevention of mucositis from radio- and chemotherapy of the head and neck. Therefore, we also evaluated the possible adverse and beneficial effects on the individual’s health regarding administration. In the end, we established that the adverse effects are practically zero, and the beneficial effects on the individual involve the reestablishment of intestinal flora and even the reestablishment of immunity against cancer cells in the individual through a mechanism of T lymphocyte stimulation.

## Figures and Tables

**Table 1:** Search strategy.

***PubMed*** (“probiotics”) AND (“oral mucositis”)
***Web of Science*** ((ALL = (probiotics)) AND ((ALL = (oral mucositis))
***Lilacs*** “probiotics” (palavras) AND “oral mucositis” (palavras)

**Table 2:** Main characteristics of studies included in the present systematic review.

Author	Year	Sample	Type of Therapy	Criteria	Results of Therapy
Jiang et al. [25]	2018	99 Patients: 64 probiotic 35 placebo	Cisplatinum plus radiotherapy 70 Gy	National Cancer Institute’s Common Terminology Criteria for Adverse Events	Reduces the severity of OM through changes in the gut microbiome
De Sanctis et al. [26]	2019	68 Patients: 32 probiotic 36 placebo	Cisplatinum plus radiotherapy 68 Gy	National Cancer Institute’s Common Terminology Criteria for Adverse Events	No difference between the two groups
Mirza et al. [27]	2022	46 Patients: 23 probiotic 23 placebo	Radiotherapy with or no chemotherapy 60–70 Gy	National Cancer Institute’s Common Terminology Criteria for Adverse Events	Reduces the severity of OM

## Data Availability

Data sharing not applicable.

## Supplementary Materials

The following supporting information can be downloaded at: https://www.mdpi.com/article/10.3390/ph16050654/s1, File S1: PRISMA Checklist.

## Abbreviations

HSCT	hematopoietic cell transplantation
IMRT	intensity modulated radiotherapy
OM	oral mucositis
LB	lactobacillus
CCRT	radiotherapy
CCRT-p	radiotherapy and probiotics
RT	radiotherapy
TLR	toll-like receptor
LTA	lipoteichoic acid
FDA	food and drug administration
IND	investigation new drug
AHRQ	Agency for Healthcare Research and Quality

## References

[1] Zhao R., Hu H., Wang Y., Lai W., Jian F. (2021). Efficacy of Probiotics as Adjunctive Therapy to Nonsurgical Treatment of Peri-Implant Mucositis: A Systematic Review and Meta-Analysis. Front. Pharmacol..

[2] Maiorana C., Beretta M., Grossi G.B., Santoro F., Herford A.S., Nagursky H., Cicciù M. (2011). Histomorphometric Evaluation of Anorganic Bovine Bone Coverage to Reduce Autogenous Grafts Resorption: Preliminary Results. Open Dent. J..

[3] Meurman J.H., Stamatova I. (2007). Probiotics: Contributions to Oral Health. Oral Dis..

[4] Gupta G. (2011). Probiotics and Periodontal Health. J. Med. Life.

[5] Gungor O.E., Kirzioglu Z., Kivanc M. (2015). Probiotics: Can They Be Used to Improve Oral Health?. Benef. Microbes.

[6] Meurman J.H., Stamatova I.V. (2018). Probiotics: Evidence of Oral Health Implications. Folia Med..

[7] Bonifait L., Chandad F., Grenier D. (2009). Probiotics for Oral Health: Myth or Reality?. J. Can. Dent. Assoc..

[8] Mann S., Park M.S., Johnston T.V., Ji G.E., Hwang K.T., Ku S. (2021). Isolation, Characterization and Biosafety Evaluation of Lactobacillus Fermentum OK with Potential Oral Probiotic Properties. Probiot. Antimicrob. Proteins.

[9] Alqahtani F., Alshaikh M., Mehmood A., Alqhtani N., Alkhtani F., Alenazi A. (2022). Role of Probiotics for the Treatment of Peri-Implant Mucositis in Patients with and Without Type 2 Diabetes Mellitus. J. Oral Implantol..

[10] Mehta V., Sarode G.S., Obulareddy V.T., Sharma T., Kokane S., Cicciù M., Minervini G. (2023). Clinicopathologic Profile, Management and Outcome of Sinonasal Ameloblastoma—A Systematic Review. J. Clin. Med..

[11] Herford A.S., Lu M., Akin L., Cicciù M. (2012). Evaluation of a Porcine Matrix with and without Platelet-Derived Growth Factor for Bone Graft Coverage in Pigs. Int. J. Oral Maxillofac. Implant..

[12] Homayouni Rad A., Pourjafar H., Mirzakhani E. (2023). A Comprehensive Review of the Application of Probiotics and Postbiotics in Oral Health. Front. Cell Infect. Microbiol..

[13] Sayardoust S., Johansson A., Jönsson D. (2022). Do Probiotics Cause a Shift in the Microbiota of Dental Implants—A Systematic Review and Meta-Analysis. Front. Cell Infect. Microbiol..

[14] Temelci A., Yılmaz H.G., Ünsal G., Uyanik L.O., Yazman D., Ayali A., Minervini G. (2023). Investigation of the Wetting Properties of Thalassemia Patients’ Blood Samples on Grade 5 Titanium Implant Surfaces: A Pilot Study. Biomimetics.

[15] Gugnacki P., Sierko E. (2021). Is There an Interplay between Oral Microbiome, Head and Neck Carcinoma and Radiation-Induced Oral Mucositis?. Cancers.

[16] Nguyen T., Brody H., Radaic A., Kapila Y. (2021). Probiotics for Periodontal Health-Current Molecular Findings. Periodontol. 2000.

[17] Cicciù M., Herford A.S., Stoffella E., Cervino G., Cicciù D. (2012). Protein-Signaled Guided Bone Regeneration Using Titanium Mesh and Rh-BMP2 in Oral Surgery: A Case Report Involving Left Mandibular Reconstruction after Tumor Resection. Open Dent. J..

[18] Shu Z., Li P., Yu B., Huang S., Chen Y. (2020). The effectiveness of probiotics in prevention and treatment of cancer therapy-induced oral mucositis: A systematic review and meta-analysis. Oral Oncol..

[19] Das M. (2019). Probiotics for chemoradiotherapy-induced oral mucositis. Lancet Oncol..

[20] Contaldo M., Di Stasio D., Romano A., Fiori F., Vella F.D., Rupe C., Lajolo C., Petruzzi M., Serpico R., Lucchese A. (2022). Oral candidiasis and novel therapeutic strategies: Antifungals, phytotherapy, probiotics, and photodynamic therapy. Curr. Drug Deliv..

[21] Cheng B., Zeng X., Liu S., Zou J., Wang Y. (2020). The efficacy of probiotics in management of recurrent aphthous stomatitis: A systematic review and meta-analysis. Sci. Rep..

[22] Liu Y.-C., Wu C.-R., Huang T.-W. (2022). Preventive Effect of Probiotics on Oral Mucositis Induced by Cancer Treatment: A Systematic Review and Meta-Analysis. Int. J. Mol. Sci..

[23] Cereda E., Caraccia M., Caccialanza R. (2018). Probiotics and mucositis. Curr. Opin. Clin. Nutr. Metab. Care.

[24] Page M.J., McKenzie J.E., Bossuyt P.M., Boutron I., Hoffmann T.C., Mulrow C.D., Shamseer L., Tetzlaff J.M., Akl E.A., Brennan S.E. (2021). The PRISMA 2020 statement: An updated guideline for reporting systematic reviews. BMJ.

[25] Jiang C., Wang H., Xia C., Dong Q., Chen E., Qiu Y., Su Y., Xie H., Zeng L., Kuang J. (2019). A randomized, double-blind, placebo-controlled trial of probiotics to reduce the severity of oral mucositis induced by chemoradiotherapy for patients with nasopharyngeal carcinoma. Cancer.

[26] De Sanctis V., Belgioia L., Cante D., La Porta M.R., Caspiani O., Guarnaccia R., Argenone A., Muto P., Musio D., De Felice F. (2019). *Lactobacillus brevis CD2* for Prevention of Oral Mucositis in Patients with Head and Neck Tumors: A Multicentric Randomized Study. Anticancer Res..

[27] Mirza M.A., Aruna D., Irukulla M. (2022). Efficacy of *Bacillus clausii* UBBC-07 spores in the amelioration of oral mucositis in head and neck cancer patients undergoing radiation therapy. Cancer Treat. Res. Commun..

[28] Gupta N., Ferreira J., Hong C.H.L., Tan K.S. (2020). Lactobacillus reuteri DSM 17938 and ATCC PTA 5289 ameliorates chemotherapy-induced oral mucositis. Sci. Rep..

[29] Maria-Aggeliki K., Nikolaos K., George K., Vassilis K., Kyrias G. (2009). The Potential Clinical Impact of Probiotic Treatment for the Prevention and/or Anti-Inflammatory Therapeutic Effect Against Radiation Induced Intestinal Mucositis. A Review. Recent Pat. Inflamm. Allergy Drug Discov..

[30] Lu Y., Luo X., Yang D., Li Y., Gong T., Wang J., Li B., Cheng J., Chen R., Guo X. (2022). Effects of probiotic supplementation on related side effects after chemoradiotherapy in cancer patients. Front. Oncol..

[31] Araujo L.D.C., Furlaneto F.A.C., da Silva L.A.B., Kapila Y.L. (2022). Use of the Probiotic *Bifidobacterium animalis* subsp. *lactis* HN019 in Oral Diseases. Int. J. Mol. Sci..

[32] Yeung C.Y., Chan W.T., Jiang C.B., Cheng M.L., Liu C.Y., Chang S.W., Chiang Chiau J.S., Lee H.C. (2015). Amelioration of Chemotherapy-Induced Intestinal Mucositis by Orally Administered Probiotics in a Mouse Model. PLoS ONE.

[33] Huang L., Chiau J.S.C., Cheng M.L., Chan W.T., Jiang C.B., Chang S.W., Yeung C.Y., Lee H.C. (2019). SCID/NOD mice model for 5-FU induced intestinal mucositis: Safety and effects of probiotics as therapy. Pediatr. Neonatol..

[34] Cappello F., Rappa F., Canepa F., Carini F., Mazzola M., Tomasello G., Bonaventura G., Giuliana G., Leone A., Saguto D. (2019). Probiotics Can Cure Oral Aphthous-Like Ulcers in Inflammatory Bowel Disease Patients: A Review of the Literature and a Working Hypothesis. Int. J. Mol. Sci..

[35] Prisciandaro L.D., Geier M.S., Butler R.N., Cummins A.G., Howarth G.S. (2011). Evidence Supporting the use of Probiotics for the Prevention and Treatment of Chemotherapy-Induced Intestinal Mucositis. Crit. Rev. Food Sci. Nutr..

[36] Jayaraj R., Kumaraswamy C., Shetty S., Raymond G., Govind S.K., Chandramoorthy H.C., Shaw P. (2020). Clinical approaches to interpreting the findings of systematic review and meta-analysis of the effectiveness of probiotics in the prevention and treatment of Cancer Therapy-Induced Oral Mucositis (CTIOM). Oral Oncol..

[37] Li D., Li Q., Liu C., Lin M., Li X., Xiao X., Zhu Z., Gong Q., Zhou H. (2014). Efficacy and safety of probiotics in the treatment of *Candida*-associated stomatitis. Mycoses.

[38] Sangild P.T., Shen R.L., Pontoppidan P., Rathe M. (2018). Animal models of chemotherapy-induced mucositis: Translational relevance and challenges. Am. J. Physiol. Gastrointest. Liver Physiol..

[39] Ozen M., Dinleyici E.C. (2015). The history of probiotics: The untold story. Benef. Microbes.

